# Effects of Sitting and Supine Positions on Tongue Color as Measured by Tongue Image Analyzing System and Its Relation to Biometric Information

**DOI:** 10.1155/2024/1209853

**Published:** 2024-03-23

**Authors:** Aya Murakami, Akira Morita, Yuki Watanabe, Takaya Ishikawa, Toshiya Nakaguchi, Sadayuki Ochi, Takao Namiki

**Affiliations:** ^1^Center for Pharmaceutical Education, Faculty of Pharmacy, Yokohama University of Pharmacy, 601 Matano-Cho, Totsuka-Ku, Yokohama 245-0066, Japan; ^2^Sumida Kampo Clinic, East Asian Medicine Center, Chiba University Hospital, 1-19-1 Bunka, Sumida-Ku, Tokyo 131-0044, Japan; ^3^Department of Japanese-Oriental (Kampo) Medicine, Graduate School of Medicine, Chiba University, 1-8-1 Inohana, Chuo-Ku, Chiba 260-8670, Japan; ^4^Graduate School of Engineering, Chiba University, 1-33 Yayoi-Cho, Inage-Ku, Chiba 263-8522, Japan; ^5^Center for Frontier Medical Engineering, Chiba University, 1-33 Yayoi-Cho, Inage-Ku, Chiba 263-8522, Japan

## Abstract

Tongue diagnosis is one of the important diagnostic methods in Kampo (traditional Japanese) medicine, in which the color and shape of the tongue are used to determine the patient's constitution and systemic symptoms. Tongue diagnosis is performed with the patient in the sitting or supine positions; however, the differences in tongue color in these two different positions have not been analyzed. We developed tongue image analyzing system (TIAS), which can quantify tongue color by capturing tongue images in the sitting and supine positions. We analyzed the effects on tongue color in two different body positions. Tongue color was quantified as *L*^*∗*^*a*^*∗*^*b*^*∗*^ from tongue images of 18 patients in two different body positions by taking images with TIAS. The CIEDE 2000 color difference equation (Δ*E*_00_) was used to assess the difference in tongue color in two different body positions. Correlations were also determined between Δ*E*_00_, physical characteristics, and laboratory test values. The mean and median Δ*E*_00_ for 18 patients were 2.85 and 2.34, respectively. Of these patients, 77.8% had a Δ*E*_00_ < 4.1. A weak positive correlation was obtained between Δ*E*_00_ and systolic blood pressure and fasting plasma glucose. Approximately 80% of patients' tongue color did not change between the sitting and supine positions. This indicates that the diagnostic results of tongue color are trustworthy even if medical professionals perform tongue diagnosis in two different body positions.

## 1. Introduction

Traditional medicine is now one of the most important treatment options, used by about 80% of developing countries and half of developed countries [1–8]. Among them, TCM, especially acupuncture, is reported by the WHO to be the most widely practiced traditional medicine in the world [9]. Against this background, traditional medicine (Kampo medicine, TCM, and Korean medicine, which originated from ancient Chinese medicine and have common diagnostic methods) is attracting widespread interest not only in East Asian countries but also all over the world. However, many challenges exist regarding the standardization of diagnostic methods, diagnostic support, education of medical professionals involved in diagnosis, and differences in diagnostic results among medical professionals for traditional medicine.

One of the diagnostic methods used in Kampo medicine, TCM, and Korean medicine is tongue diagnosis. Tongue diagnosis is a noninvasive diagnostic method and involves observation of the patient's tongue color (light white, light red, red, deep red, and purple), tongue coating (white, white-yellow, yellow, brown, gray, and black), and shape [10]. Tongue findings can provide information on the patient's constitution, blood circulation, water metabolism, and other systemic physiological/pathological conditions [11–19]; therefore, tongue diagnosis can lead to the selection of the appropriate prescriptions for the patients.

However, the position of the patient during tongue diagnosis is not uniform. Some physicians and pharmacists observe the tongue in the sitting position, while others observe the tongue in the supine position to obtain the patient's abdominal findings [20, 21]. Acupuncturists often observe the tongue in the supine position because of acupuncture treatment [22, 23]. Furthermore, the patient position for tongue diagnosis varies according to national and regional medical culture and customs. Therefore, a factor to be considered in the standardization of diagnosis is not only quantification but also clarification of the influence of the patient position on tongue diagnosis.

The patient's body position also could affect the diagnostic outcome in modern medicine. For example, the supine position is known to cause changes in gravity and circulating plasma volume compared to the sitting position, resulting in lower values of blood cell components, hemoglobin, albumin, and total protein [24, 25]. In Kampo medicine, the impression that the thickness of the diameter of the sublingual vein varies with body position has been described clinically, and it has been pointed out that the supine position may lead to overestimation of the severity of the patient's condition in Kampo medicine based on the diameter of the sublingual vein [26, 27]. Thus, it is important to objectively and quantitatively clarify the influence of different body positions on diagnostic results. However, the relationship between body position (sitting vs. supine) and tongue color has not been reported.

Tongue diagnosis is affected by the observation environment, such as light source and room temperature, and it requires knowledge and experience acquired through years of practice [28]. To overcome this issue, we developed the tongue image analyzing system (TIAS) to further promote tongue diagnosis and support objective diagnosis [29–33].

TIAS is equipped with an integrating sphere and diffused illumination to maintain a constant light intensity. This allows for stable conditions that are not affected by environmental factors such as light source, dryness, or room temperature; in addition, it is possible to capture tongue images without gloss imaging artifacts of the tongue. Furthermore, from the captured tongue image, it is possible to quantify the diameter of the sublingual vein and the tongue color values using the *L*^*∗*^*a*^*∗*^*b*^*∗*^ color system defined by the Commission internationale de l'éclairage (CIE).

In this study, TIAS was used to quantify tongue color in the sitting and supine positions, and the effects of different body positions on tongue color were examined.

## 2. Methods

### 2.1. Subjects

A retrospective study was conducted on 18 patients (7 male and 11 female) who visited the Department of Kampo Medicine at Chiba University Hospital between May and August 2017. The Department provided sufficient explanation, and patient consent was obtained at the time of the initial visit.

This study was approved by the Ethics Committee of Chiba University (Approval No. 812), and anonymity was assured.

### 2.2. Study Design

#### 2.2.1. Tongue Imaging in the Sitting and Supine Positions

**Figure 1:**
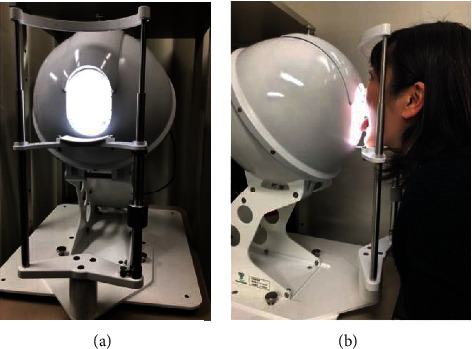
Tongue image analyzing system (TIAS) for the sitting position. (a) Appearance of TIAS for the sitting position. (b) Tongue imaging in the sitting position.

**Figure 2:**
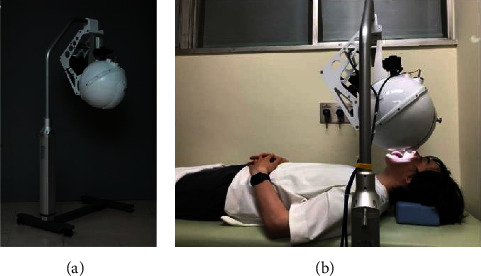
Tongue image analyzing system (TIAS) for the supine position. (a) Appearance of TIAS for the supine position. (b) Tongue imaging in the supine position.

TIAS was used for tongue imaging. The conventional TIAS (Figure 1) was designed to capture the tongue in the sitting position, and we developed a second TIAS that can capture the tongue in the supine position (Figure 2). Functions such as lighting are the same as for the TIAS in the sitting position [24], but a lifting function was added. The procedure was to capture the tongue with TIAS for the sitting position and then capture the tongue with TIAS for the supine position. All patients had their tongues photographed within approximately 3 mins after being placed in the supine position.

#### 2.2.2. Quantification of Tongue Color Value by TIAS

Tongue color values were converted from RGB to *L*^*∗*^*a*^*∗*^*b*^*∗*^ color space data defined by the CIE based on tongue photographs taken with the TIAS and used for analysis [11]. *L*^*∗*^*a*^*∗*^*b*^*∗*^ color space is a device-independent color space and a color system consisting of *L*^*∗*^ for lightness (brightness to darkness), *a*^*∗*^ for chromaticity (redness [+*a*] to greenness [−a]), and *b*^*∗*^ for chromaticity (yellowness [+*b*] to blueness [−b]).

**Figure 3:**
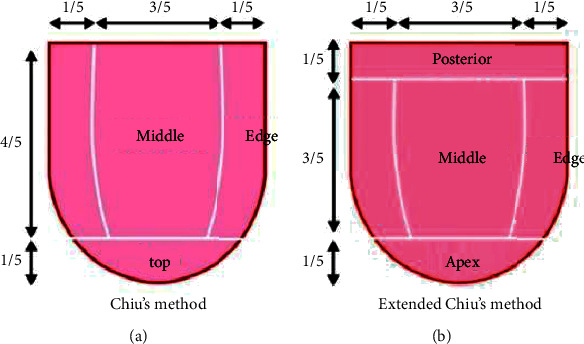
Extraction method for tongue color values. (a) Chiu's method. (b) Extended Chiu's method.

The tongue fractionation method proposed by Chiu divides the tongue into three regions: edge portion, middle portion, and top portion [34]. Compared to the 5-point method [23], Chiu's method has a larger area for each analysis region and is more robust to noise and area shifts for each analysis. Each region is defined by a ratio based on the tongue shape as shown in Figure 3(a). However, Chiu's method does not define the posterior tongue in the organ segmentation method, making it difficult to correspond with the results of the 5-point method. In this study, we proposed an extension to Chiu's method in which the division ratio is adjusted and the posterior tongue is added to Chiu's conventional method. As a result, four regions can be obtained: tongue edge, tongue posterior, tongue middle, and tongue apex, as in the 5-point method. Each region was defined by a ratio based on the tongue shape, as shown in Figure 3(b).

In this study, the *L*^*∗*^*a*^*∗*^*b*^*∗*^ values of the tongue edge, which are least affected by tongue coating, were used in the analysis.

#### 2.2.3. Comparison of Tongue Color Values by Color Difference

The CIE DE 2000 color difference formula (Δ*E*_00_) was used for the color difference of tongue color values in the sitting and supine positions. The conventionally used color difference CIE LAB (Δ*E*^*∗*^*ab*) has a problem in that the calculated value does not match the human visual evaluation, and the CIE DE 2000 color difference formula was proposed as an alternative to Δ*E*^*∗*^*ab* [35]. Δ*E*_00_ is a quantitative index of the color difference between two points in the CIE *L*^*∗*^*a*^*∗*^*b*^*∗*^ space and is calculated by the following formula based on the lightness difference (Δ*L*′), saturation difference (Δ*C*′), and hue difference (Δ*H*′), with correction using weighing coefficients (SL, SC, and SH) and parametric coefficients (kL, kC, and kH), as shown below. In this study, kL : kC : kH = 1 : 1 : 1.

(1) $$\begin{array}{c} \Delta E_{00}=\sqrt{{\left(\frac{\Delta L^{\prime }}{k_{L}∙S_{L}}\right)}^{2}+{\left(\frac{\Delta C^{\prime }}{k_{C}∙S_{C}}\right)}^{2}+{\left(\frac{\Delta H^{\prime }}{k_{H}∙S_{H}}\right)}^{2}+\left(R_{T}\left(\frac{\Delta C^{\prime }}{k_{C}∙S_{C}}\right)\left(\frac{\Delta H^{\prime }}{k_{H}∙S_{H}}\right)\right)}\\ \Delta L^{\prime }=L*\text{supine}\;\text{position}-L*\text{sitting}\;\text{position}\\ \Delta C^{\prime }=C^{\prime }\text{supine}\;\text{position}-C^{\prime }\text{sitting}\;\text{position}\\ \Delta H^{\prime }=2\sqrt{C^{\prime }\text{sitting}\;\text{position}\;C^{\prime }\text{supine}\;\text{position}}\;\sin\left(\frac{\Delta h^{\prime }}{2}\right). \end{array}$$

Δ*E*_00_ is widely used in dentistry for teeth [36–38], the gingiva [39–41], and the tongue [42, 43], and thresholds such as 50 : 50% perceptibility threshold (PT) and 50 : 50% acceptability threshold (AT) are frequently used to make decisions. 50 : 50% PT is the threshold at which 50% of observers notice the color difference between two samples while the remaining 50% of observers cannot notice the difference. Similarly, 50 : 50% AT is the threshold at which 50% of observers can accept the color difference [38]. Besides AT = 4.1 [40, 44, 45], which is frequently used as 50 : 50% AT in gingival color studies, AT = 4.0 [39] and AT = 2.8 [39] have been reported. In this study, we apply AT = 4.1, which is often used in studies of gingival color, as an approximation of tongue color. Δ*E*_00_ < 4.1 was an acceptable color difference, and the percentage was determined.

#### 2.2.4. Search for Factors Associated with Δ*E*_00_

To identify factors associated with the color difference between the sitting and supine positions, we sought correlations of Δ*E*_00_ with physical characteristics (age, sex, body mass index [BMI], smoking status, blood pressure, and blood test values).

Furthermore, based on the diagnostic criteria for hypertension, dyslipidemia, and diabetes mellitus, we examined the presence or absence of laboratory test items above reference value (blood pressure grade II and III: systolic blood pressure [SBP] ≥ 160 mmHg or diastolic blood pressure [DBP] ≥ 100 mmHg [46], triglycerides ≥500 mg/dL [47], blood glucose ≥126 mg/dL [48], and HbA1c ≥ 6.5% [49]) for which prompt consultation is recommended.

### 2.3. Statistical Analysis

As correlations between Δ*E*_00_ and physical characteristics, *η*^2^ correlation ratios were obtained for gender and smoking, and *r* correlation coefficients were obtained for the other items. All statistical analyses were performed using JMP (v14, SAS Institute, Cary, NC), with *P* < 0.05 indicating a significant difference.

## 3. Results

The median age and BMI of the 18 patients were 74.0 years and 24.3 kg/m^2^, respectively (Table 1). The Δ*E*_00_ of these patients in the sitting and supine positions was <4.1 in 14 (77.8%) patients (Table 2). The mean and median Δ*E*_00_ values of the 18 patients (2.85 and 2.34, respectively) were below 4.1.

Analysis of correlations with Δ*E*_00_ showed weak positive correlations with blood glucose, HbA1c, and SBP (Table 3).

The criteria for each disease were applied to the patients in this study to determine whether any items corresponded to the recommended level of consultation. In patients with Δ*E*_00_ < 4.1, 13 (92.9%) patients in 14 patients had either no items or only one applicable item.

**Figure 4:**
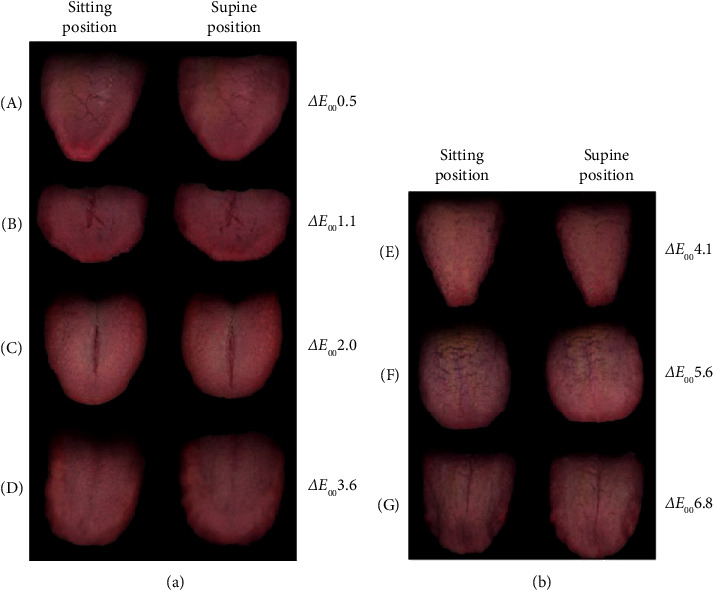
Seven cases with a Δ*E*_00_ of 0.5 (minimum) to 6.8 (maximum). (a) Sitting position. (b) Supine position.

However, in patients with Δ*E*_00_ ≥ 4.1, three of the four patients had two or more applicable items (Table 1). Furthermore, the patient with the highest Δ*E*_00_ (Patient No. 4) had three applicable items. Seven tongue images are shown, ranging from a minimum Δ*E*_00_ 0.5 to a maximum Δ*E*_00_ 6.8 as shown in Figure 4.

## 4. Discussion

This study quantitatively analyzed tongue color measured in different body positions; moreover, this is the first report to examine the effect of body position on tongue color. Diagnosis in Kampo medicine is based on the five senses, supported by experience and knowledge, and the information obtained from the sense of sight is very important. To avoid subjective bias, we quantified tongue color values with TIAS and calculated color differences.

Changes in body position are known to affect laboratory test results during blood sampling and blood pressure measurement because of the effects of gravity and changes in circulating plasma volume. For this reason, the need for standardization of patient positioning during blood samplings and measurements has been pointed out [30, 31]. However, our analysis revealed that approximately 80% of patients are unaffected by positional differences between the sitting and supine positions, at least for the diagnosis of tongue color. This indicates that the position of the patient during the tongue diagnosis does not affect the diagnostic results not only between doctors, who diagnose patients in the sitting or supine position, but also between medical professionals, such as acupuncturists, who are presumed to be mainly in the supine position, and pharmacists, who are presumed to be mostly in the sitting position.

On the other hand, tongue color difference for approximately 20% of patients exceeded Δ*E*_00_ 4.1. Lifestyle-related diseases such as hypertension, dyslipidemia, and diabetes mellitus have been reported to be associated with decreased deformability, increased red blood cell viscosity, and decreased blood fluidity in the microcirculation [50, 51]. We focused on the association between tongue color and laboratory test values in relation to lifestyle-related diseases. We found a weak correlation between Δ*E*_00_ and three laboratory test values (SBP, HbA1c, and FPG), and higher values of these tests were associated with greater color differences by body positions.

Furthermore, when each patient's laboratory test items were determined based on the diagnostic criteria for each disease, 13 of 14 patients (92.9%) with Δ*E*_00_ < 4.1 had either none or only one item applicable, whereas 3 of 4 patients with Δ*E*_00_ ≥ 4.1 had 2 or more items applicable (Table 1). 4 patients were taking antihypertensive drugs and hypoglycemic drugs including insulin but were poorly controlled. Accordingly, if several laboratory test values such as blood pressure, HbA1c, and fasting plasma glucose are far out of the normal range, we have to consider poor microcirculatory, which could be a factor affecting tongue color in tongues with large numbers of capillaries.

In this study, the tongue was photographed approximately 3 min after the patient was placed in the supine position. In clinical practice, tongue diagnosis is often performed after the patient has been placed in the supine position and has had their pulse and abdomen assessed. The imaging time is generally considered to reflect the clinical situation by taking into account the diagnostic process. Furthermore, we quantified the change in tongue color over time in the supine position. We observed that the change in tongue color values from 3 to 20 min after assuming the supine position was small and within the acceptable color difference range (Supplemental Figure 1 and Supplemental Table 1). Therefore, it is estimated that there is no effect on tongue diagnosis due to differences in position until about 20 min after lying in the supine position. However, the effect on tongue color for patients who are in the supine position for extended periods of time, such as during hospitalization, is uncertain. In addition, we were unable to identify the factors for the one patient with a Δ*E*_00_ ≥ 4.1, even though only one of the laboratory test items was determined to be above the reference value.

Based on the results of the present study, tongue color values in patients whose laboratory test values such as blood pressure and blood glucose level were normal were not affected by the two different body positions. This suggests that in clinical practice, tongue color tends to be visually equivalent regardless of which position tongue diagnosis is performed, and this could mean that the diagnostic results also tend to be the same. On the other hand, factors related to lifestyle-related diseases such as blood pressure, HbA1c, and fasting plasma glucose showed a weak correlation with tongue color. This suggests that in patients with deteriorating physiological and/or pathological conditions, the position of the patient during tongue diagnosis may affect tongue color.

TIAS combines quantification, objectivity, and reproducibility. We believe that this study contributes to the understanding of traditional medicine including Kampo medicine and the development of integrative medicine through the elucidation of the relationship between pathological conditions and tongue color, education of medical professionals, standardization of tongue diagnosis, and support for tongue diagnosis.

## Figures and Tables

**Table 1:** Patients' characteristics (*n* = 18).

No.	Age (year)	Sex (M/F)	BMI (kg/m^2^)	Smoking (yes/no)	CVD drug (yes/no)	SBP (mmHg)	DBP (mmHg)	TG (mg/dL)	HDL-C (mg/dL)	LDL-C (mg/dL)	HbA1c (%)	FPG (mg/dL)
1	84	F	27.8		Yes	120	40	179	60	123	6.3	152^*∗∗*^
2	77	F	29.3			138	68	117	71	129	7.0^*∗∗*^	103
3	71	M	22.2	Yes		129	73	49	49	134	6.1	132^*∗∗*^
4	83	M	21.4	Yes	Yes	176^*∗∗*^	86	68	60	88	7.2^*∗∗*^	168^*∗∗*^
5	77	F	26.2		Yes	122	80	201	64	132	6.2	128^*∗∗*^
6	40	F	29.9	Yes		129	80	154	48	183^*∗∗*^	6.8^*∗∗*^	121
7	73	M	24.2	Yes		133	76	185	43	111	7.7^*∗∗*^	140^*∗∗*^
8	85	M	21.4			160^*∗∗*^	80	74	80	128	5.6	98
9	82	M	24.3	Yes		136	89	66	60	114	5.4	102
10	75	F	25.5		Yes	120	65	117	40	141	6.3	132
11	57	F	21.3		Yes	99	63	43	69	120	4.9	92
12	76	M	19.5	Yes		128	72	115	64	134	7.1^*∗∗*^	160^*∗∗*^
13	80	F	25.4		Yes	145	80	190	46	111	5.7	91
14	65	F	25.4		Yes	137	82	58	63	112	6.4	115
15	64	F	26.1			148	80	96	54	182^*∗∗*^	5.8	100
16	56	F	21.4			126	78	276	53	229^*∗∗*^	5.7	99
17	69	M	22.3	Yes		114	68	97	101^*∗∗*^	100	5.5	96
18	44	F	16.5	Yes		88	51	144	53	113	5.5	88

BMI: body mass index; CVD: cardiovascular disease; SBP: systolic blood pressure; DBP: diastolic blood pressure; TG: triglyceride; HDL-C: high-density lipoprotein cholesterol; LDL-C: low-density lipoprotein cholesterol; FPG: fasting plasma glucose. ^*∗∗*^Items exceeding diagnostic reference value.

**Table 2:** Color difference between the sitting and supine positions (*n* = 18).

No.	(*L*^*∗*^, *a*^*∗*^, *b*^*∗*^)		Δ*L*′	Δ*C*′	Δ*H*′	Δ*E*_00_
	Sitting position	Supine position				
1	(52.5, 28.6, 4.2)	(54.0, 29.6, 6.6)	1.47	1.46	2.26	2.1
2	(41.6, 29.4, 3.2)	(40.4, 30.2, 2.4)	−1.20	0.82	−0.93	1.3
3	(40.5, 25.5, 0.7)	(41.1, 29.3, 4.0)	0.58	4.43	3.02	2.7
4	(36.0, 23.9, 2.4)	(43.5, 25.1, 5.3)	7.49	1.78	2.64	6.8
5	(44.5, 29.8, 4.5)	(47.7, 31.5, 7.6)	3.24	2.35	2.70	3.7
6	(46.1, 22.2, 6.7)	(45.6, 24.3, 6.2)	−0.46	2.22	−1.09	1.3
7	(42.1, 20.5, 2.7)	(47.6, 23.3, 4.7)	5.51	3.67	1.50	5.6
8	(38.6, 30.0, 5.8)	(39.3, 27.6, 4.1)	0.70	2.74	1.26	1.5
9	(35.9, 28.2, 3.0)	(35.2, 30.9, 4.1)	−0.67	2.95	0.80	1.4
10	(47.3, 26.1, 8.3)	(45.9, 29.0, 9.6)	−1.41	3.36	0.42	2.0
11	(46.4, 28.9, 10.1)	(43.8, 30.5, 9.8)	−2.60	1.43	−0.73	2.6
12	(43.0, 24.2, 5.7)	(38.6, 26.5, 5.0)	−4.38	2.43	−1.17	4.1
13	(38.8, 32.5, 6.9)	(36.5, 34.3, 7.2)	−2.27	1.88	−0.07	2.1
14	(42.1, 28.0, 8.7)	(38.2, 30.3, 8.2)	−3.94	1.89	−0.98	3.6
15	(50.0, 27.3, 8.3)	(43.9, 29.4, 7.5)	−6.09	2.01	−1.39	6.1
16	(41.2, 29.4, 5.0)	(40.6, 31.4, 5.8)	−0.60	2.29	0.42	1.1
17	(42.1, 23.6, 3.5)	(44.5, 26.2, 4.1)	2.36	3.04	0.24	2.5
18	(44.6, 25.3, 4.8)	(44.3, 26.1, 5.2)	−0.30	1.02	0.17	0.5

*L* ^*∗*^ for lightness (brightness to darkness), *a*^*∗*^ for chromaticity (redness [+*a*] to greenness [−*a*], and *b*^*∗*^ for chromaticity (yellowness [+*b*] to blueness [−*b*)]). Δ*L*′: lightness difference, Δ*C*′: chromaticity difference, Δ*H*′: hue difference, Δ*E*_00_: color difference.

**Table 3:** Correlation between Δ*E*_00_ and patient findings (*n* = 18).

Items	*r*	*η* ^2^	*p* value
Age (year)	−0.28		0.26
Sex (M/F)		<0.1	0.95
BMI (kg/m^2^)	−0.02		0.95
Smoking (yes/no)		<0.1	0.56
CVD drug (yes/no)	0.04		0.43
SBP (mmHg)	−0.49		0.04
DBP (mmHg)	−0.35		0.16
TG (mg/dL)	−0.19		0.44
HDL-C (mg/dL)	−0.08		0.74
LDL-C (mg/dL)	−0.18		0.47
HbA1c (%)	−0.49		0.04
FPG (mg/dL)	−0.55		0.02

BMI: body mass index; CVD: cardiovascular disease; SBP: systolic blood pressure; DBP: diastolic blood pressure; TG: triglyceride; HDL-C: high-density lipoprotein cholesterol; LDL-C: low-density lipoprotein cholesterol; FPG: fasting plasma glucose; *r*: correlation coefficient; *η*^2^: correlation ratio.

## Data Availability

The original contributions presented in the study are included in the article/Supplementary Material, and further inquiries can be directed to the corresponding author/s.

## Supplementary Materials

Supplementary Materials Supplementary Figure 1: we observed the change in tongue color values from 3–20 min after assuming the supine position was small and within the acceptable color difference range. Therefore, it is estimated that there is no effect on tongue diagnosis due to differences in position until about 20 min after lying in the supine position. Supplementary Table 1: change in Δ*E*_00_ at 20 min after the supine position (*n* = 5).
