# Supplementary material for: Effects of Sitting and Supine Positions on Tongue Color as Measured by Tongue Image Analyzing System and Its Relation to Biometric Information
Source: Evid Based Complement Alternat Med. 2024 Mar 23;2024:1209853. doi: 10.1155/2024/1209853 (PMC10981547; doi:10.1155/2024/1209853)
Supplement: Supplementary Materials — Supplementary Figure 1: we observed the change in tongue color values from 3–20 min after assuming the supine position was small and within the acceptable color difference range. Therefore, it is estimated that there is no effect on tongue diagnosis due to differences in position until about 20 min after lying in the supine position. Supplementary Table 1: change in ΔE00 at 20 min after the supine position (n = 5). [file 1209853.f1.zip › 20240304_supplemental_table.docx]

**Supplemental Table.** Change in *ΔE_00_* at 20 min after the supine position (n=5)

| No. | Sex  (M/F) | Age  (year) | *ΔE_00_* | | | |
| --- | --- | --- | --- | --- | --- | --- |
|  |  |  | 3 min | 5 min | 10 min | 20 min |
| 1 | M | 34 | 0 | 0.97 | 1.01 | 0.37 |
| 2 | F | 33 | 0 | 0.94 | 3.31 | 1.22 |
| 3 | F | 23 | 0 | 0.91 | 2.02 | 1.28 |
| 4 | M | 27 | 0 | 1.87 | 0.89 | 1.86 |
| 5 | M | 42 | 0 | 1.24 | 2.71 | 2.37 |

*ΔE_00_*: difference in color.
